# A qualitative exploration of factors influencing site of delivery (home, public or private hospital) in three North Karnataka districts as described by pregnant women, mothers of neonates, husbands and grandmothers

**DOI:** 10.1186/1753-6561-6-S5-P7

**Published:** 2012-09-28

**Authors:** Sharon Bruce, Kaveri Gurav, Andrea Blanchard, Anu Roy, HL Mohan, BM Ramesh, Lisa Avery

**Affiliations:** 1 University of Manitoba, Winnipeg, Canada; 2 Karnataka Health Promotion Trust, Bangalore, India

## Introduction

The National Rural Health Mission (NHRM) aims to increase the uptake of safe institutional delivery among rural communities in Karnataka. Previous studies in India have found that while there has been increasing numbers of institutional deliveries, those who had lower socio-economic status, were from scheduled caste (SC), or had less education or media exposure were less likely to use hospitals for delivery. A few studies on quality of care consistently found a more positive perception and experience at private hospitals. The purpose of this study was to explore the factors influencing choice of birthing site, specifically home, public and private hospitals, and the decision-making processes involved in the choice, in North Karnataka.

## Methods

In the context of the Karnataka Heath Promotion Trust’s (KHPT) maternal, neonatal and child health (MNCH) program, 112 qualitative interviews were conducted among pregnant women, mothers of neonates (≤ 30 days), grandmothers and husbands in three North Karnataka districts: Bagalkot, Bellary and Gulbarga. Local residents with previous research experience were hired and trained to conduct the interviews. Interviews were completed between October and December 2010. Interviews were conducted in local languages and then translated to *Kannada* and English for analysis. Thematic analysis was undertaken in which codes were identified through review of transcripts and categories were created based on the codes. Comparative analysis was completed looking for similarities and differences in experiences, perceptions and decision-making by birthing site, participant type, district and sociodemographic characteristics. Ethics approval was obtained from St. John’s College, Bangalore and the University of Manitoba Human Research Ethics Board, Winnipeg, Canada.

## Results

Of the 112 participants just over half were living below the poverty line (54%), belonged to scheduled caste (42%), and were uneducated (55%). A greater proportion of pregnant women and grandmothers were uneducated and below poverty than the new mothers and husbands. While some participants indicated that hospital delivery is becoming more favoured among the younger generation, others described many factors that dissuaded them from institutional delivery. First, some perceived or experienced poor quality of care and poorly equipped and unclean facilities, particularly at public hospitals. In addition, some participants reported having to pay for supplies and/or schemes at public hospitals. While private hospitals, in general, were seen as delivering high quality services some cited the high cost for private hospital delivery as prohibitive. Having a ‘normal’ delivery was highly valued among all participants, therefore many participants offered that the common practice of performing Caesarean sections and assisted deliveries at both public and private hospitals was a concern and factored into their decisions about delivery site. Practices associated with ‘normal’ birth, such as hot water, dietary preferences were more likely to be available at private versus public hospitals. However, some preferred practices that were available at home births were not available at either hospital type. Finally, though some received the benefits of incentive schemes or aid from Accredited Social Health Activists (ASHA), many others did not.

## Discussion

The perceptions of poor quality of care and facilities at government institutions in north Karnataka among the study respondents appears to play a significant role in discouraging public institutional delivery; the influence of these perceptions is perhaps even more than actual experience of. This indicates a need to address negative perceptions and actual quality of hospital care, particularly that of government hospitals in poor areas. In addition, perceptions of institutional delivery may be improved by reducing the number of medically unnecessary C-sections, as well as ensuring consistent coverage by schemes and comprehensive outreach to marginalised communities by ASHAs.

## Competing interests

None declared.

## Funding statement

The study was funded by Bill and Melinda Gates Foundation.

